# Staunton, Virginia

**Published:** 1891-03

**Authors:** 


STAUNTON, VIRGINIA; ITS PAST, PRESENT, AND FUTURE. By Armistead C. Gordon, Esq. With illustrations from photographs by Edmund Berkley. New York: The South Publishing Co., 76 Park Place, 1891.

This beautiful pamphlet of seventy-six pages, issued by the Staunton Development Co., is intended to set forth the advantages of Staunton, Va., as a desirable place to settle.

Staunton is one of the most important cities of the "New South." It is located in the famous Shenandoah Valley, and is surrounded by coal and iron mines and coke ovens. To still further advance and improve this locality is the mission of the "Staunton Development Co." They have, therefore, issued the pamphlet now under notice. It is filled with fine illustrations, copied from photographs, consisting of views of the surrounding country, of the mines, public buildings, private residences, and the fine hotel, built in the latest style and called Hotel Altemonte. For information address D. Z. Evans, Jr., agent, Room 41, Frederick Brown Building, Fifth and Chestnut Streets, Philadelphia, Pa.

